# Non-equidistant scanning approach for millimetre-sized SPM measurements

**DOI:** 10.1186/1556-276X-7-213

**Published:** 2012-04-11

**Authors:** Petr Klapetek, Miroslav Valtr, Petr Buršík

**Affiliations:** 1 Czech Metrology Institute, Okružní 31, Brno, 638 00, Czech Republic; 2 Faculty of Informatics, Masaryk University, Botanická 68a, Brno, 602 00, Czech Republic

## Abstract

Long-range scanning probe microscope (SPM) measurements are usually extremely time consuming as many data need to be collected, and the microscope probe speed is limited. In this article, we present an adaptive measurement method for a large-area SPM. In contrast to the typically used line by line scanning with constant pixel spacing, we use an algorithm based on several levels of local reﬁnement in order to minimize the amount of information that would be useless in the data processing phase. The data obtained from the measurement are in general formed by *xyz *data sets that are triangulated back with a desired local resolution. This enables storing more relevant information from a single measurement as the data are interpolated and regularized in the data processing phase instead of during the measurement. In this article, we also discuss the inﬂuence of thermal drifts on the measured data and compare the presented algorithm to the standard matrix-based measuring approach.

## Background

Many novel industrial components, e. g. optoelectronic devices, microchips, diﬀraction gratings or photonic crystals, are typically formed by highly integrated microstructures arranged on very large areas. The inspection and control of such components are problematic as we need to detect small imperfections on large areas or to evaluate small diﬀerences between many semiconductor mask critical dimensions. Scanning probe microscopy techniques (e. g. atomic force microscopy) can be used for this task if suﬃciently large areas can be scanned. The bottleneck of using scanning probe microscopes was always the scanning range; however today, specialized equipment can be found in the literature and even on the market [1-5] featuring very small uncertainties (less than 1 nm) over very large volumes. However, even if the range of microscopes has changed, the maximum speed of the tip is still nearly the same. Large-scale measurements are therefore very slow.

**Figure 1:**
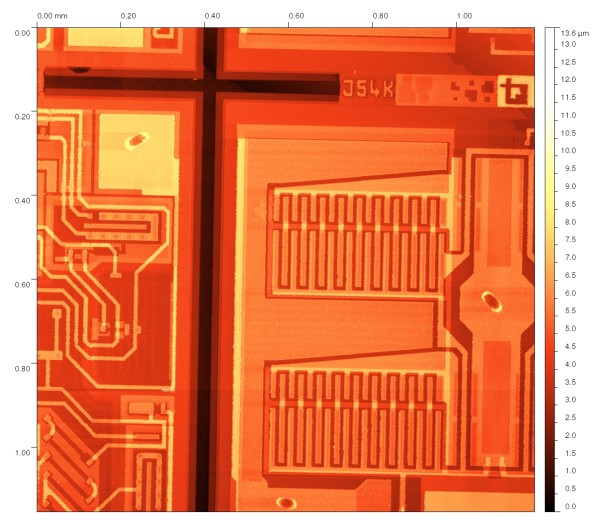
**Large-area AFM measurement**. Typical large-area measurement of the microchip surface using the atomic force microscope technique. The lateral dimensions in *x *and *y *axes are approximately 1, 187 *μ*m.

Even if we measure on very large areas, like those shown on Figure 1 for a microchip surface, only a very small part of the measured data is ﬁnally used for the evaluation. From Figure 1, we would probably measure several critical dimensions on observed interconnections only. The rest of the data would be left aside even if it took several hours to acquire them and even if they occupy hundreds of megabytes of disk space. This is a large waste of eﬀort. One of the possibilities how to overcome this is to measure data in a non-equidistant approach (not creating a regular matrix of points during measurements). If we are able to measure the overall sample geometry with low resolution and local features that will be used for surface properties evaluation with high resolution, we can both speed up the measurement and reduce the need for data storage.

In this article, we discuss one possible realization of a non-equidistant measurement method for a long-range scanning probe microscope and discuss its applications for diﬀerent samples typical for microelectronics and solar cell industry.

## Methods

### Experimental arrangement

**Figure 2:**
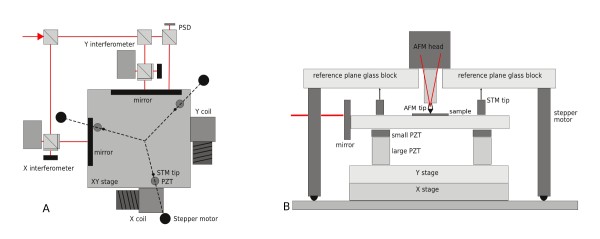
**Long-range SPM schematics**. **(A) ***xy *Motion system (position of three reference plane sensors and actuators shown as well) and **(B) **reference plane system schematics (two-dimensional plot, note that in practice the system has a threefold symmetry as seen from the top view in part A).

A home-built long-range scanning system was used for the implementation and testing of the adaptive scanning algorithm [6]. It is based on commercial crossed-roller bearing stages combined with piezoceramic actuators used to compensate the imperfections of the bearing mechanism. Three interferometers are used for all three-axis translation monitoring and feedback. For stage rotation monitoring (axis normal to sample surface), an autocollimator is used. For non-planarity compensation and two more axis rotation compensations (axes parallel to sample surface), an optical quality reference plane and a set of tunneling current sensors are used. The atomic force microscope head was used for determining the probe-sample distance and performing the *z*-axis motion (based on piezoceramic actuators). The construction of the system is illustrated in Figure 2 and in more detail described in [6]. The thermal stability of the system is assured using an active stabilization box with digitally controlled, thermally stabilized water.

For illustrative measurements, three different samples were used: a calibration grating that is usually used for commercial scanning probe microscopes calibration, a microchip surface and a solar cell surface with a typical pyramidal structure used for light trapping. These represent typical measurands requesting large-scale measurements combined with high-resolution details. Contact mode measurements were performed using standard contact tips from Nanosensors (PPP-CONTR series, NANOSENSORS, Rue Jaquet-Droz 1, Case Postale 216, CH-2002 Neuchatel, Switzerland).

### Adaptive measurement algorithm

There are numerous ways how to perform measurements of a rectangular surface area. Logically, the most straightforward and mostly used approach is to measure in a rectangular grid mapping pixel to pixel and directly creating the image. Even if this is the most convenient scanning approach and the data can be processed very easily after measurement, it leads to a large loss of data already during the scanning process. As the microscope needs to maintain the feedback loop during the motion of the tip, the amount of the collected data in a single proﬁle is usually much larger than the number of pixels of the resulting image (e. g. by an order or more). Data are then resampled to the requested number of pixels, loosing the high-resolution information already acquired in between them. A natural improvement of 'standard' square scanning probe microscope (SPM) image would therefore be a square set of data with non-rectangular pixel size, where fast axis spacing would be much smaller than the one of the slow axis. Implementing such a scanning method could already dramatically improve the amount of information collected in a single scan (in the same time), even if the method is still quite a regular one.

On the other side, adaptive sampling could be realized as forming a completely random distribution of points in the *xy *plane. Based on some surface properties, like local roughness, we could measure the data forming a completely irregular set of points that would be triangulated afterwards. However, as the microscope in principle measures high-resolution data over some continuous path, this could be ineﬀective. It is therefore desirable to measure a set of proﬁles (not necessarily forming a Cartesian grid).

In order to simplify the problem, we started implementing a scanning algorithm based on acquiring rows and columns of diﬀerent length, not organized in a regularly spaced matrix. The prerequisite of the presented iterative process is to decide what should be the ﬁnal *xy *axes desired resolution and what is the *z*-axis precision we want to reach. The key points of the reﬁnement algorithm are as follows:

1. Measure a net formed by rows and columns with coarse *xy *resolution and interpolate data to the ﬁnal resolution.

2. Measure and add an interleaved net of rows and columns to form a data with resolution which is twice as ﬁne and interpolate data to the ﬁnal resolution.

3. Identify subsets between rows and columns where the interpolated data between the last two iterations diﬀer more than by a requested *z *precision criterion.

4. Where the *z *precision criterion failed, measure a net of rows and columns with twice as ﬁne resolution on those rectangles. Note that in order to save the measurement time, this process needs to be optimized so that the movement between diﬀerent reﬁnement areas is minimized. An optimum path for the SPM probe is therefore planned merging all the necessary movements (including movements between different areas) with the criterion of the shortest total distance. This is in principle a traveling salesman problem, and here, it is solved using the nearest neighbor algorithm.

**Figure 3:**
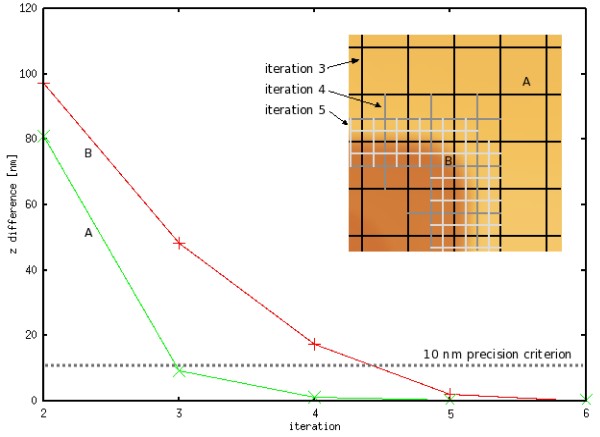
**Adaptive measurement process illustration**. The graph shows the convergence of the *z *value of a typical point on a ﬂat sample area (point A) and close to a steep slope (point B) if the iterative reﬁnement process is not stopped by the precision criterion. The precision criterion is used to stop reﬁnements locally as shown in the inset.

**Figure 4:**
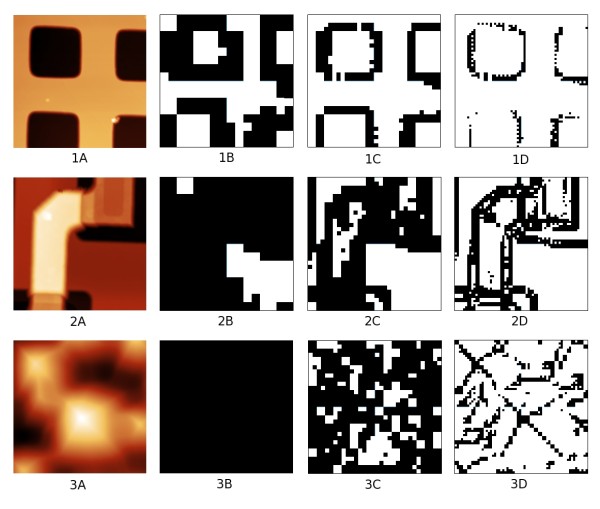
**Local reﬁnement process on diﬀerent surfaces**. Topography of (1) 2D calibration grating, (2) microchip surface and (3) solar cell front surface together with areas where the *z *precision criterion failed and a reﬁnement needs to be planned (black color) for three successive levels of iterative process (A, B, C).

The whole reﬁnement process is illustrated in Figure 3. If the surface exhibits regular areas (e. g. a microchip surface), areas measured in each reﬁnement iteration become smaller and smaller, up to a point where all data are measured with the requested z precision. In Figure 4, measured areas are shown for a calibration grating, a microchip surface and a solar cell light-trapping surface. Areas where the *z *precision criterion failed are marked by black for the three levels of reﬁnement. In these areas, a reﬁnement in next iteration is planned and performed. It should be noted that for a completely random surface (white noise), the ﬁnal number of data measured is twice larger than for a standard matrix measurement (we measure both rows and columns independently). However, as the surfaces are typically locally continuous, we usually need to measure a much smaller number of data points than for a standard matrix measurement. Moreover, the suggested algorithm could in principle lead to missing a small structure in case that it would be missed in the ﬁrst two iterations. The only solution to overcome this is not to use too coarse initial grid spacing.

When we have the data measured, we cannot save them as a regular matrix, so we use a general set of *xyz *values for storing the data. Algorithms available for SPM data processing however expect a regular matrix in most of the cases, and so do also the vast majority of software packages available for SPM data processing and analysis. The easiest way on how to overcome this is to regularize the data back before analysis - now with the desired resolution that can be locally much larger than for the coarse image. We can therefore perform zooms in the measured data - and where the data are densely sampled, we can obtain high-resolution details. For interpolation purposes, data are triangulated by a fast divide and conquer routine (similar to the one described in [7]) reaching optimal worst-case complexity of *O*(*n *log *n*), where n is the number of data used for triangulation. Recursive divisions alternate between horizontal and vertical cuts. A Delaunay triangulation and a Voronoi diagram are created using this approach. With Delaunay triangulation and Voronoi diagram computed, we can use a number of interpolation methods. At the moment, a simple planar interpolation of the triangulated data is utilized.

The presented algorithm was implemented using libraries of Gwyddion open source software for SPM data analysis http://gwyddion.net. While the scanning routines can hardly be shared to the public (these are connected to the microscope hardware), we expect that the tested triangulation and regularization approach will be made available to Gwyddion users as part of our future work.

## Results and discussion

The suggested measurement algorithm has three main beneﬁts compared to the matrix-based scanning methods:

1. It can save the amount of necessary measured information while measuring with very high resolution, thus shortening the measurement time and minimizing the probe wear while preserving the necessary resolution on critical surface structures.

2. It allows the user to perform further local reﬁnements easily while the sample is still in the microscope, not necessarily based on the algorithm criteria but also on the user's requirements.

3. It can automatically measure the data allowing the user to perform zooms *offline*, e. g. in the data processing phase after the measurement. This can be useful namely for automated image processing or inspection purposes.

In order to document the effect of the reduction of the necessary scanned path, we compare the path length saved by the presented algorithm to the standard matrix-based approach for the surface shown in Figure 3. The resulting total tip path reductions for two diﬀerent ﬁnal accuracies are summarized in Table 1. It can be noted that the more reﬁnement levels are performed the more data are saved. This is however not valid generally but only on regular structures like the one presented here. As stated above, for completely random data, we cannot save anything using the reﬁnement algorithm.

**Table 1:** Performance of the measurement algorithm

Accuracy	20 nm	3 nm
Grating	18%	34%
Microchip	42%	54%
Solar cell	41%	68%

This table summarizes the total tip path reduction for two diﬀerent ﬁnal accuracies (20 and 3 nm). Note that 100% corresponds to the path needed for obtaining the regularly sampled data with the same accuracy.

**Figure 5:**
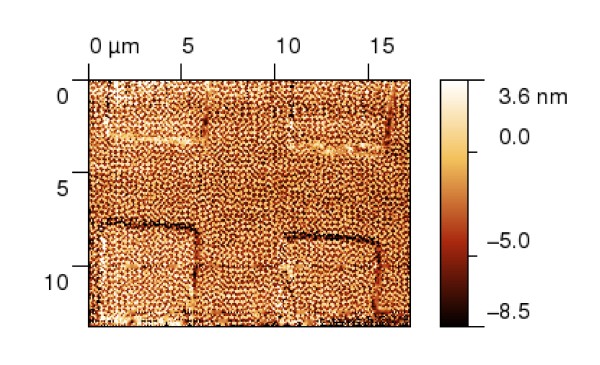
**Comparison of the matrix algorithm and the presented algorithm**. This ﬁgure shows the result of a subtraction of images obtained using the two algorithms. Note that the local diﬀerences are caused only by relatively high noise of present long-range system *z *scanner and slight mismatch between the two images (due to a slight shift caused by retract and approach between the two measurements, the images had to be cropped and aligned manually).

In order to display the real performance of the algorithm if implemented in the microscope, we compare the results of the matrix algorithm and the presented algorithm on real data of a calibration grating. The same ﬁnal resolution was requested. In Figure 5, we can see the result of the subtraction of images obtained using the two algorithms. We can see that the local diﬀerences are caused only by relatively high noise of the presently used long-range system z scanner and a slight mismatch between the two images (due to a slight shift caused by retraction and approach between the two measurements, the images had to be cropped and aligned manually).

It should be noted that the obtained data have diﬀerent statistical properties than a typical matrix-based SPM image. There is no diﬀerence between the fast and slow axes as both *x *and *y *directions are used for the measurement. It has no sense therefore to distinguish between directions while evaluating direct or statistical quantities. All parasitic eﬀects, such as drift or noise, inﬂuence data almost isotropically. This could be understood as a drawback in some sense; on the other side, it can simplify the treatment of uncertainties while obtaining proﬁles of different orientation with respect to the main axes or 2D statistical properties evaluation.

**Figure 6:**
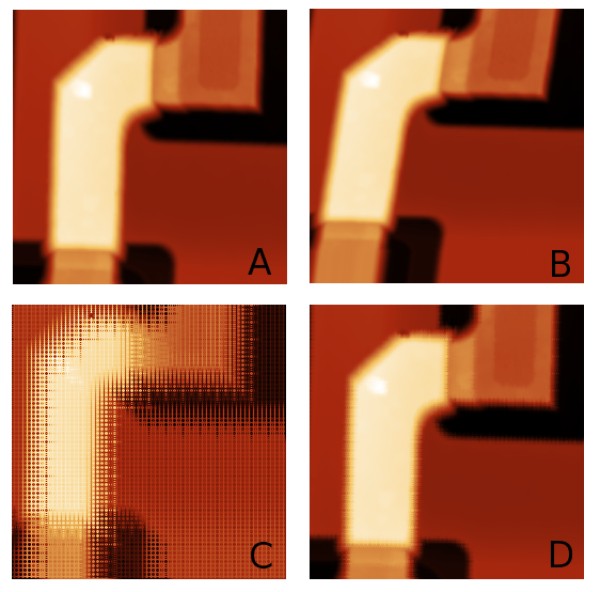
**Drift inﬂuence**. Microchip surface **(A) **and inﬂuence of general xyz drift to standard matrix-based measurement process **(B)**, iterative reﬁnement **(C) **and measurement process and iterative reﬁnement process after drift guess from the ﬁrst two iterations and successive drift compensation **(D)**.

An important issue is the inﬂuence of drift on the measurement process. Drift, namely of thermal origin, can be observed in many SPM systems [8,9]. In a typical matrix-based SPM image, drift is mainly seen in the slow scanning axis, leading to image distortion as seen in Figure 6B. Note that the coordinate system origin in this simulation is in the top left corner of the image. Based on the analysis of the known surface structures or based on repetitive scans, we can determine the drift rate, which is usually a time-dependent decaying function, with a maximum right after the instrument start-up or a sample exchange [10]. If we use the scanning approach presented in this work, the data in the image are not measured successively, so the straightforward drift determination from AFM image is not possible as the drift inﬂuences interleaved values signiﬁcantly (see Figure 6C). However, drift can still be evaluated. As in each reﬁnement level, we measure on the same area as was already measured in the previous iteration, and we even obtain data at exactly the same points (at row/column crossings); we can determine the drift rate already during reﬁnement process for areas that are being reﬁned. We can do this using the following approach:

• Create an interpolated image from one reﬁnement level (using the standard procedure from the previous section);

• Create an interpolated image from the next level skipping the data measured in the previous level;

• Use cross-correlation to determine the shift between the two data sets (in all three axes), which are the *x *and *y *drift values; and

• Shift one data set according to the cross-correlation result and subtract the two data sets, which gives us the *z *drift value.

As an illustration of the process, we have simulated data measurement with a constant drift vector of (3, 3, 0.3) nanometres per second in Figure 6. We have used the part of the microchip surface seen in Figure 6A, using already measured data (without observable drift) and adding the drift during the simulated measurement (all movements were performed with the same velocity). Drift was evaluated from levels 1 and 2 of the reﬁnement process where, for this sample, we still measure on nearly the whole area (the local reﬁnement criterion still holds everywhere). Using the above-mentioned process, we have evaluated the drift vector as (3.3 ± 0.5, 2.7 ± 0.3, 0.29 ± 0.15) nanometres per second which is a good estimate of the drift rate. In Figure 6D, a simulated measurement with correction based on this estimated drift rate is shown (for drift estimation, the ﬁrst two iterations were used), obviously leading to signiﬁcant correction of the image. Of course, if the drift rate is not constant, the above-mentioned approach would not be an optimal one; however, the user could repeat it after several reﬁnement iterations to correct the drift rate estimate. Generally, it can be seen that the drift leads to a much more evident image distortion when our adaptive reﬁnement algorithm is used. On the other side, even the data obtained in a regular matrix approach are inﬂuenced by the drift, and if we do not know the measured structure properties, we need to use a similar correlation technique to determine the drift. Data are therefore 'wrong' in both cases, but in the regular matrix case, they look better and allow the user to ignore the systematic errors caused by the drift. The large inﬂuence of the drift on the presented algorithm could therefore be seen even as a beneﬁt for a metrologist - if the systematic error is quantitatively the same in both cases anyway, the adaptive approach prevents the fact that it would rest hidden in the data.

## Conclusion

We have implemented an adaptive reﬁnement measuring algorithm in a long-range scanning probe microscope. For surfaces with large regular areas, the use of this adaptive scanning instead of a regular matrix scanning can save the time necessary for the measurement and enable high-resolution zooming in the measured data. This can be interesting namely when measuring large areas of diﬀerent manufactured nano-and micro-structures, like electronic parts, photonic crystals, diﬀraction gratings, etc. Using the presented approach, we iteratively measure with higher and higher resolution on critical surface areas, determined by a simple and universal criterion. In the data processing phase, the set of *xyz *data obtained from the measurement is triangulated, and necessary details are regularized in order to reach requested resolution. This enables the user to perform zooming in the data processing phase, with no need of performing further measurements.

## Competing interests

The authors declare that they have no competing interests.

## Authors' contributions

PK carried out the development of the adaptive measurement algorithm. MV performed atomic force microscopy measurements on long-range SPM, and PB wrote software routines for general *xyz *data triangulation. All authors read and approved the ﬁnal manuscript.
